# Content of Phenolic Acids as a Marker of Polish Honey Varieties and Relationship with Selected Honey-Quality-Influencing Variables

**DOI:** 10.3390/antiox11071312

**Published:** 2022-06-30

**Authors:** Anna Puścion-Jakubik, Elżbieta Karpińska, Justyna Moskwa, Katarzyna Socha

**Affiliations:** Department of Bromatology, Faculty of Pharmacy with the Division of Laboratory Medicine, Medical University of Białystok, Mickiewicza 2D Street, 15-222 Białystok, Poland; elzbieta.karpinska@umb.edu.pl (E.K.); justyna.moskwa@umb.edu.pl (J.M.); katarzyna.socha@umb.edu.pl (K.S.)

**Keywords:** honeybee, buckwheat honey, Poland, markers, phenolic acids

## Abstract

Phenolic acids are an important component of honey. Literature data indicate their pro-health properties and diversified content in different varieties. Therefore, the aim of our study was to evaluate the content of phenolic acids in bee honey. The material for the research was 49 samples of honey obtained from beekeepers from Poland. Selected phenolic acids were determined by HPLC with PDA detection. Additionally, total phenolic content (TPC), color intensity, color on the Pfund scale, water content, electrical conductivity, and FRAP were assessed. A higher trans-ferulic acid content is accompanied by a stronger free radical scavenging ability. It was shown that buckwheat honeys are characterized by a high TPC value (196.59 mg GAE/100 g), color intensity (2109.2 mAU), color on the Pfund scale (159.8 mm Pfund), and high activity in the FRAP assay (0.403 equivalent of µmol Fe^2+^/mL). The median obtained in the DPPH test for this honey variety was 41.1%. Moreover, the highest median of 4-hydroxybenzoic acid (3.129 mg/100 g) in buckwheat honey was shown. Buckwheat honeys have promising antioxidant properties and should be included in diets low in antioxidants.

## 1. Introduction

Bee honey is a product of very diverse composition. It includes, among others, sugar compounds, water, proteins, organic acids, vitamins, minerals, phenolic compounds, enzymes, and many other ingredients [1]. Honey available for sale should be properly labeled, including, inter alia, the name of the variety. Beekeepers define a variety based on the color, consistency, smell, taste or on the basis of observation of the plants from which the bees collect nectar or honeydew.

Earlier publications indicate that a large percentage of honey is incorrectly labeled [2]. The classic method for determining the type of honey is the melissopalinological method, which consists of counting pollen grains under a microscope and classifying them into botanical species. This is a time-consuming method that requires detailed observation of the grains. Sometimes, it is emphasized that its results are ambiguous and difficult to interpret. Therefore, other methods of identifying honey varieties are being sought. For example, an electronic potentiometric tongue has been developed to help identify the honey variety [3]. The nuclear magnetic resonance (NMR) method was used to distinguish between nectar and honeydew honey [4]. Another method that can be used to identify honey varieties is the method of fluorescence spectroscopy. It was used to distinguish, among others, acacia, linden, and sunflower honey [5]. 

Other methods of honey classification are based on searching for characteristic markers or identifying fingerprints. For example, high-performance liquid chromatography with diode array detection and tandem mass spectrometry (HPLC-DAD-MS/MS) was used to distinguish between chaste honey and rape honey. The following markers were considered: ferulic acid, kaempferol, and morin. Additionally, chromatographic fingerprints at 270 nm and 360 nm were identified. The above methods were used in conjunction with chemometric techniques [6]. An attempt to identify the honey variety on the basis of its antioxidant properties was also undertaken by Dżugan et al. (2018). Buckwheat honey had the strongest antioxidant properties, and rape honey had the weakest [7].

In addition, the health-promoting properties of bee honey may be conditioned by the presence of compounds with antioxidant properties, including phenolic acids. The literature describes many beneficial properties of bee honey, including its use in the treatment of burns and ulcers [8], rosacea [9], acute cough [10], and bedsores [11]. HPLC is one of the most popular methods used to determine the content of individual compounds with antioxidant properties [12].

Phenolic substances, which are phenol derivatives, are synthesized by plants. They are divided into simple phenols and polyphenols. Polyphenols contain more than one hydroxyl in their molecule structure. Polyphenols can exist in free form or in combination with other substances, such as glycosides (made of aglycone and sugar residue). Phenolic acids include compounds derived from cinnamic and benzoic acids, including caffeic acid, ferulic acid, *p*-hydroxybenzoic acid, *p*-coumaric acid, protocatechuic acid, syringic acid, and vanillic acid. They can interact with biologically active molecules and protect them against damage [13]. In addition to phenolic acids, the antioxidant properties of honey are due to, among others, flavonoids, vitamins (such as vitamins C and E), and minerals (including zinc and manganese) [1]. For example, the literature data indicate that the most common flavonoids in acacia honeys are: apigenin, chrysin, galangin, genistein, kaempferol, luteolin, myricetin, pinobanksin, pinocembrin, and quercetin. Those characteristic of manuka honey are: chrysin, galangin, isorhamnetin, kaempherol, luteolin, pinobanksin, pinocembrin, and quercetin [14].

Therefore, the aim of the research was to assess whether selected phenolic acids can be a marker of individual varieties of honey from Poland, as well as to correlate the content of these acids with selected parameters determining the quality of the honey, such as color scale, color intensity, total phenolic content, water content, electrical conductivity, and % free radical scavenging in DPPH assay.

## 2. Materials and Methods

### 2.1. Materials

The research material consisted of 49 samples of natural bee honeys: buckwheat (*n* = 15), linden (*n* = 9), multi-flower light (*n* = 3), dandelion (*n* = 4), nectar–honeydew (*n* = 4), rape (*n* = 8), honeydew (*n* = 3), and heather (*n* = 3). Honey was purchased in Poland; each sample was made by a different beekeeper. Until analysis, the honeys were stored at 4 °C. 

All solvents were HPLC grade, and all chemicals were analytical and reagent grade. Formic acid (min. 98%) was obtained from Merck (Darmstadt, Germany). Methanol was purchased from J.T. Baker (Avantor, Gliwice, Poland).

Ultrapure water was obtained from Simplicity™185 Water Purification System (Merck Millipore, Darmstadt, Germany).

HPLC standards of polyphenols such as: 3,4-dihydroxybenzoic acid (3,4-DHBA), 4-hydroxybenzoic acid (4-HBA), caffeic acid (CA), *p*-coumaric acid (p-CA), syringic acid (SA), trans-ferulic acid (*t*-FA), vanillic acid (VA), and reagents for determining the total content of phenolic compounds (Folin–Ciocalteu reagent, Na_2_CO_3_) were obtained from Sigma–Aldrich (St. Louis, MO, USA). Individual stock solutions of each analyte and a mixture of them were prepared in methanol. 

### 2.2. Methods

#### 2.2.1. Identification of the Varieties of Honey

The classification of variety was made on the basis of melissopalinological analysis, in accordance with the Regulation of the Minister of Agriculture and Rural Development [15]. From each honey, 10 g was weighed in a centrifuge tube, supplemented with 50 °C water to 20 mL, mixed, and centrifuged for 10 min at 3000 rpm. The precipitate was decanted, water was added again, and centrifuged. When the sediment was about 0.1 cm, a layer of water of about 0.5 ml was left above it, and when it was about 0.3 cm - a layer of about 1 ml of water was left, a homogeneous suspension was obtained and applied to a microscope slide. At least two microscopic preparations were made of each honey, in which pollen grains were classified to botanical varieties. On the basis of the grains present in the greatest predominance in given bee honey, each was given a variety name.

#### 2.2.2. Determination of Water Content

Honey in an amount of 5 g was weighed into a test tube, closed with a stopper, and placed in a water bath from 45 °C until brought to a liquid state. Then, a few drops of honey were placed in the refractometer, and the refractive index was read. In the case of temperatures above 20 °C, the factor was increased by 0.00023/1 °C, and in the case of temperatures below 20 °C, it was reduced in a similar manner. Then, the water content was read from the table in the Regulation. For each honey, at least 2 determinations were made. The results are expressed in % [15].

#### 2.2.3. Determination of Electrical Conductivity

Based on the water content of each honey, the amount to be weighed was calculated according to the following formula:

$$M=\frac{20\;g\times 100}{MS}$$,

where:

*M*—the mass of honey to be weighed (g),

*MS*—dry matter content, calculated as the difference between 100% and the water content, expressed as %.

The honey was weighed out and made up to 100 mL with distilled water. The conductivity cell was rinsed with the sample, and a honey solution (40 mL) was placed in a water bath at a temperature of 20 °C; when the temperature of the solution was 20 °C, the electrical conductivity was measured. The electrical conductivity of honey was calculated according to the formula:

S=K×G, where:

*SH*—specific conductivity of honey (mS × cm^−1^),

*K*—constant of the conductivity cell (cm^−1^),

*G*—conductivity (mS).

#### 2.2.4. Determination of Color Intensity

In order to determine the color intensity, 5 ± 0.001 g of honey was weighed, and water at 45 °C temperature was added at a volume of 10 g and mixed thoroughly. The solution was then sonicated and filtered through a 0.45 µm filter. The absorbance of the solutions was measured at 450 and 720 nm. The final result was the difference in absorbance at the two wavelengths, expressed in mAU. For each sample, three determinations were performed, and the final result was the mean result [16].

#### 2.2.5. Determination of Color on the Pfund Scale

To determine the color of natural bee honey using the Pfund scale, 5 ± 0.001 g of sample was weighed, the samples were each dissolved into 10 mL of distilled water, and they were mixed well. The samples were then placed in a water bath at 50 °C to dissolve the sugar crystals. After obtaining a clear solution, absorbance was measured at 635 nm against distilled water. The Pfund color scale was calculated using the formula:

mm Pfund=−38.70+371.39× Absorbance.

The final result is the average of three measurements [17].

#### 2.2.6. Determination of Total Phenolic Content (TPC)

The total content of phenolic compounds was determined by reaction with the Folin–Ciocalteu reagent [18]. A calibration curve was prepared using a gallic acid working solution with a concentration of 2 g/L. A 1 ± 0.001 g sample was taken from each honey. Honey was dissolved in distilled water to a volume of 10 mL and then centrifuged at 2000 rpm for 5 min. Next, 0.25 mL of supernatant was collected; then, 1.25 mL of 0.2 N Folin–Ciocalteu was added, and the sample was stirred for 5 min. Next, 1 mL of Na_2_CO_3_ solution was added, mixed, and incubated in the dark at room temperature for 2 h. The contents of each tube were then mixed, and the absorbance at 760 nm against water was measured using a Hitachi U-2001 spectrophotometer. The results are presented as the mean of 3 determinations, in mg gallic acid/100 g honey.

#### 2.2.7. Determination of Radical Scavenging Activity by DPPH Assay

The ability of bee honeys to scavenge radicals was performed on the basis of the method described by Sánchez-Moreno et al. [19]. Bee honeys were dissolved in distilled water to obtain a concentration of 1 g/mL. In total, 200 µL was taken, and 1800 µL of a DPPH solution with a concentration of 0.04 mg/mL was added. The absorbance at 517 nm was measured with a spectrophotometer U-2001 (Hitachi, Tokyo, Japan). The samples were then incubated at room temperature, protected from light, for 30 min. After the incubation period, the absorbance was measured again. The % of free radical scavenging was calculated:

$$\mathrm{DPPH}\;[\%]=[\frac{A0-A30}{A30}]\;\times \;100\%,$$

where *A*0 is the absorbance at time 0, and *A*30 is the absorbance over 30 min.

#### 2.2.8. Determination of FRAP

To perform the FRAP test, the FRAP reagent was prepared (2.5 mL of a 10 mM TPTZ solution in 40 mM HCl, 2.5 mL of 20 mM FeCl3, and 25 mL of 0.3 M acetate buffer pH 3.6) [20].

To 20 µL of honey solution, 180 µL of FRAP reagent was added, and the mixture was incubated at 37 °C for 10 min. The absorbance of the mixture was then measured at 593 nm with a plate reader (UVM 340, Biogenet, Józefów, Poland). The results are presented as the equivalent of µmol Fe^2+^/mL of the sample [20].

#### 2.2.9. Preparation of Samples for HPLC Analysis–Isolation of Phenolic Compounds

Honey samples (5 g) were dissolved in 50 mL of water (adjusted to pH 2 with HCl) until completely fluid. This solution (50 mL) was then filtered through a Sep-Pak C18 cartridge (tube type SPE, Supelclean LC-18 SPE Tubes 3 mL/500 mg, Supelco Analytical, Bellefonte, PA, USA), which was previously activated with methanol (10 mL) followed by water (10 mL). The phenolic compounds were retained on the column, whilst all sugars and other polar compounds were eluted with water, and then polyphenols were eluted with 2.5 mL of a methanol–water mixture (70%, *v*/*v*) in order to validate the efficiency of extraction SPE and similar activities dealing with standards. 

Phenolic fractions in methanol evaporated under reduced pressure (22 °C). The residue was redissolved in a mixture of distilled water and HPLC-grade methanol, in proportions such as phase (22.5 MeOH parts: 76.5 H_2_O parts: 1 CH_3_COOH parts). The prepared sample was analyzed by HPLC with photodiode array (PDA) detection. The applied extraction method enabled recovery values for analyzed compounds of higher than 85%.

#### 2.2.10. HPLC Analysis

HPLC analyses of honey extracts were performed using an Flexar HPLC system (Perkin Elmer, Waltham, MA, USA) with a photodiode array detector (PDA) and using Chromera LC-PDA software (Perkin Elmer, Waltham, MA, USA). Separations were carried out with reversed-phase column Synergi 4 µm C-18 (Merck, Darmstadt, Germany; 250 × 4.60 mm, particle size 4 micron, 80A), SecurityGuard Cartridges Fusion-RP 4 × 3.0 mm ID. A mobile phase of 22.5 MeOH:76.5 H_2_O:1 CH_3_COOH was used; a constant solvent flow rate (1 mL/min) was applied. The total analysis time was 50 min. An isocratic separation method was used using the mobile phase (22.5 MeOH:76.5 H_2_O:1 CH_3_COOH). The temperature of the column oven was set at 25 °C. The phenolic acids were detected at 254, 265, and 326 nm, since the most honey phenolic compounds show their UV absorption maxima around these three wavelengths. The comparison of UV spectra and retention times with standard compounds enabled the identification of phenolic acids presented in the analyzed honey extracts. These compounds were quantified against their external standards. The injection volume was 20 µL.

Each sample was analyzed three times, and the method was proved by repeatability test by determining peak area and retention reproducibility for different classes of compounds.

Table 1 presents data on the optimization of the method, including LOQ (limit of quantitation) and LOD (limit of detection).

The concentrations of 4-HBA, VA, and *t*-FA were read at 254 nm and 3,4-DHBA at 265 nm. However, the 326 nm wavelength was the best to read for CA, *p*-CA, and SA. During the optimization of the chromatographic conditions, the necessary quality parameters of the method were taken into account, including retention factors, relative retention factors, and resolution. The resolution of the compounds was 1.5 and above, with the exception of 3,4-DHBA, where the average resolution was 1.0–1.3.

#### 2.2.11. Statistical Analysis

Statistical analyses were performed using Statistica v.13.3 software. Values of *p* < 0.05 were considered significantly different. The correlation between all the measured parameters was evaluated using Spearman’s correlation coefficient.

In order to compare the values for several independent groups the Kuskal–Wallis ANOVA tests were performed. 

Chemometric analyzes were also performed, including cluster analysis (CA) and principal component analysis (PCA). In the CA, agglomeration was chosen as the method of grouping. The agglomeration method is single bond, and the distance measure is Euclidean distance.

## 3. Results

### 3.1. Varieties of Bee Honey

The first analytical step was to assess whether the marking of honey by beekeepers was correct in order to correctly identify the compounds present in the tested honey at a later stage. We have shown that three of the honeys labeled as ‘buckwheat’ were of a different type. None of the dandelion honeys were of this variety. Among linden honeys, an incorrect declaration of variety was found in over 56% of the honey samples. Among nectar–honeydew honeys, one out of four samples should be marked differently (Table 2).

**Figure 1:**
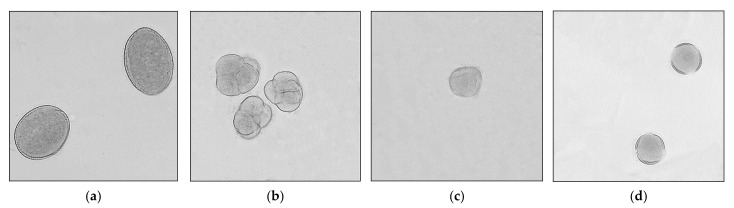
Pollen grains from honey plants: (**a**) *Fagopyrum esculentum* Moench, (**b**) *Calluna vulgaris* (L.) Hull, (**c**) *Tilia* L., (**d**) *Brassica napus* L. *var. napus*.

Figure 1 shows pictures of pollen grains characteristic of buckwheat honey (Figure 1a), for heather honey (Figure 1b), for linden honey (Figure 1c), and for rapeseed honey (Figure 1d).

### 3.2. Selected Quality Parameters

Selected quality parameters examined as part of the quality assessment and the search for markers of honey from Poland are determination of the color of honey on the Pfund scale, determination of the color intensity, total phenolic compounds (TPC), water content, and electrical conductivity (Table 3).

We showed that buckwheat honeys were characterized by the highest color value on the Pfund scale (median: 159.8 mm Pfund)—this value was significantly higher compared to the color of linden (44.9 mm Pfund), multifloral light (37.4 mm Pfund), and rape honey (84.8 mm Pfund). A similar tendency was observed for the determination of color intensity: buckwheat honey (2109.2 mAU) had the highest median. Honey of this variety was also characterized by the highest TPC value (196.59 mg GAE/100 g), as well as the highest activity in the FRAP test (0.403 equivalent of µmol Fe^2+^/mL). Interestingly, honeys of this variety have the ability to scavenge free radicals by about 41.1%. Honeydew honeys, on the other hand, showed the highest specific electrical conductivity (1.181 mS × cm^−1^), significantly higher than that of rape honey (0.242 mS × cm^−1^).

### 3.3. Profile of Phenolic Acids and the Variety of Honey

HPLC analysis showed the presence of seven phenolic compounds in honey from Poland: CA, *p*-CA, 3,4-DHEA, *t*-FA, SA, VA, and 4-HBA. 

**Figure 2:**
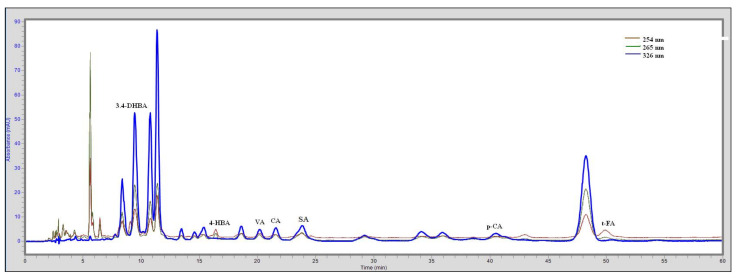
Chromatogram of the analyzed phenolic acid standards.

Figure 2 shows the chromatograms for standard substances at three wavelengths: 254 nm, 265 nm, and 326 nm.

The calculated levels of individual identified phenolic compounds in analyzed honey are shown in Table 4. The ANOVA analysis of variance showed differences in the content of individual phenolic acids between the groups. Each of the varieties of honey is characterized by a high or low content of a specific phenolic compound.

It has been shown that the content of individual phenolic compounds for varieties of honey is characteristic. 3,4-DHBA was the highest median in linden (1.993 mg/100 g) and buckwheat (1.421 mg/100 g) honey. The next compound, 4-HBA, was characteristic for buckwheat (3.129 mg/100 g) and mulitfloral dark (1.934 mg/100 g) honey. The other determined phenolic acids such as CA, VA, SA, and *t*-FA were of highest value in linden honey (1.746, 0.304, 1.107, 1.954 mg/100 g, respectively). Moreover, p-CA was of a similar level to buckwheat (0.804 mg/100 g) and mulitfloral dark (0.789 mg/100 g) honey (Table 4). 

In buckwheat honey, the highest median of 4-HBA was found—this value was significantly higher than that of the content in linden, multifloral light, nectar–honeydew, and rape. This indicates that the above compound can be considered a marker of the authenticity of buckwheat honey.

Another analyzed compound was 3,4-DHBA. Our study showed that linden honey had a significantly higher content of this phenolic acid than rape honey and CA compared to buckwheat honey.

Among the determined compounds, no characteristic concentrations were found for heather, honeydew, multifloral, nectar–honeydew, and rape honeys. 

### 3.4. Correlations

The analysis of the correlation (Table 5) between the content of phenolic compounds in honey showed a strong relationship between the content of 4-HBA and *p*-CA (r = 0.82, *p* < 0.000), between VA and SA (r = 0.60, *p* < 0.001), and between SA and CA (r = 0.51, *p* < 0.000). Among the remaining parameters, the correlation between color intensity and TPC (r = 0.90, *p* < 0.001), and color in Pfund scale and color intensity (r = 0.82, *p* < 0.001) should be emphasized. Additionally, it is worth noting the correlation between color intensity and 4-HBA (r = 0.84, *p* < 0.000).

It should be emphasized that we noted a positive correlation between the % of free radical scavenging in DPPH assay and the t-FA content (r = 0.45, *p* < 0.001).

### 3.5. Chemometric Analyzes

**Figure 3:**
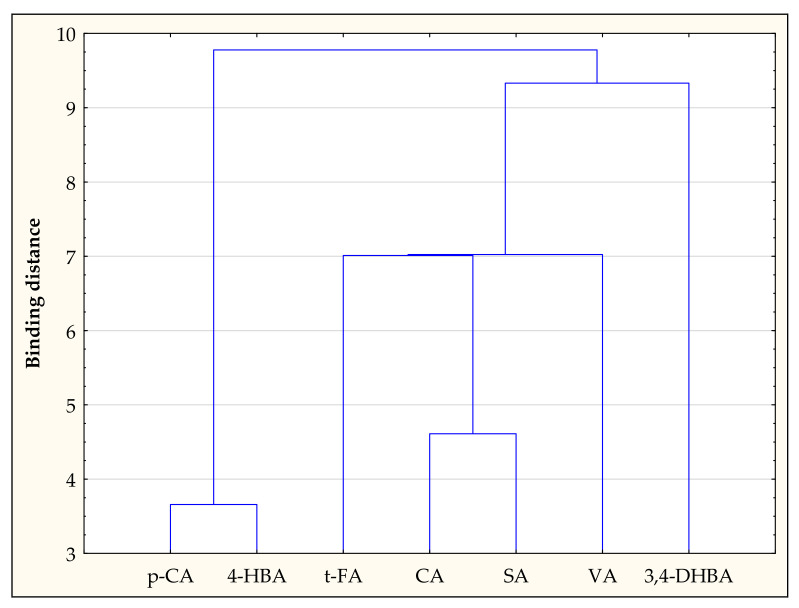
Cluster analysis for variables.

Cluster analysis performed for variables showed groups that are similar. One group was p-CA and 4-HBA, while the other group was *t*-FA, CA, SA, VA, and 3,4-DHBA (Figure 3).

**Figure 4:**
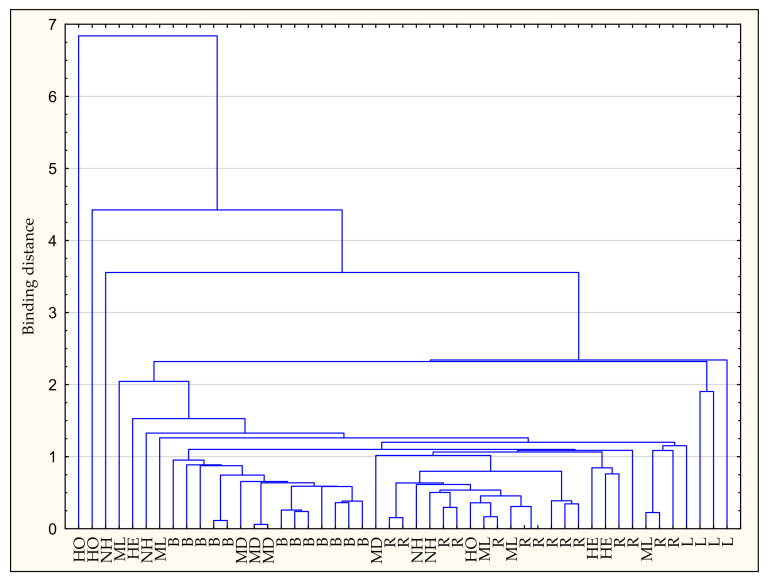
Cluster analysis for cases. B—buckwheat honey, HE—heather honey, HO—honeydew honey, L—linden honey, MD—multifloral dark honey, ML—multifloral light honey, NH—nectar–honeydew honey, R—rape honey.

The analysis carried out for the cases, based on the contents of phenolic acids, mainly distinguished honeydew honey. The focus on linden honey is also worth emphasizing. Multiflower dark honeys have also qualified for the group containing buckwheat honey (Figure 4).

Then, principal components analysis (PCA) was carried out, the purpose of which was to reduce the variables and classify the honey varieties. The first main component accounted for 43.84% of the variance; the second, 17.31%; the third, 14.45% (total 75.60%); and the subsequent components less than 10% of the variance.

**Figure 5:**
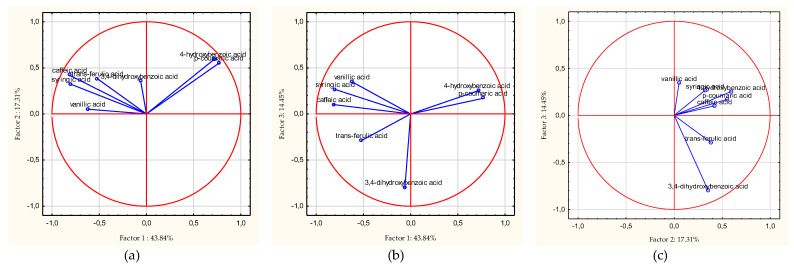
Projection of variables depending on the phenolic acids in a two – factor plane: factor 1 × factor 2 (**a**), factor 1 × factor 3 (**b**), factor 2 × factor 3 (**c**).

**Figure 6:**
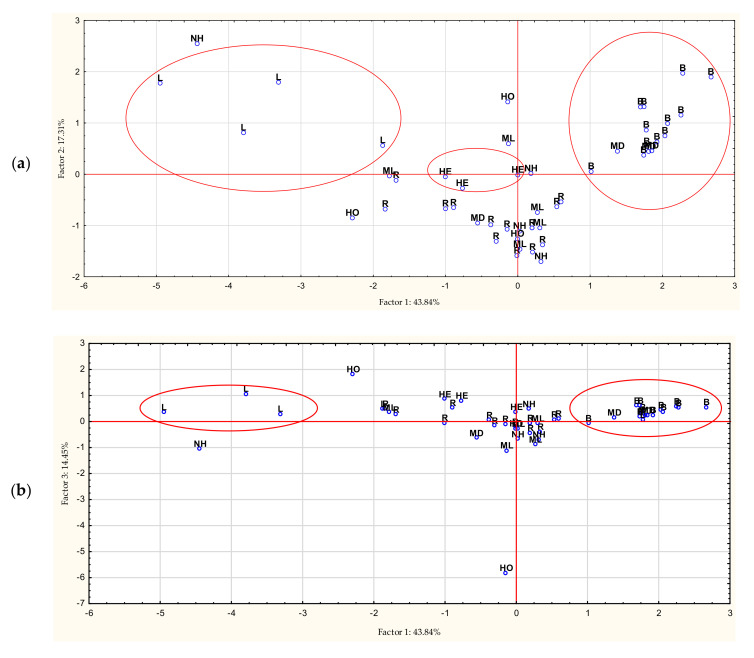

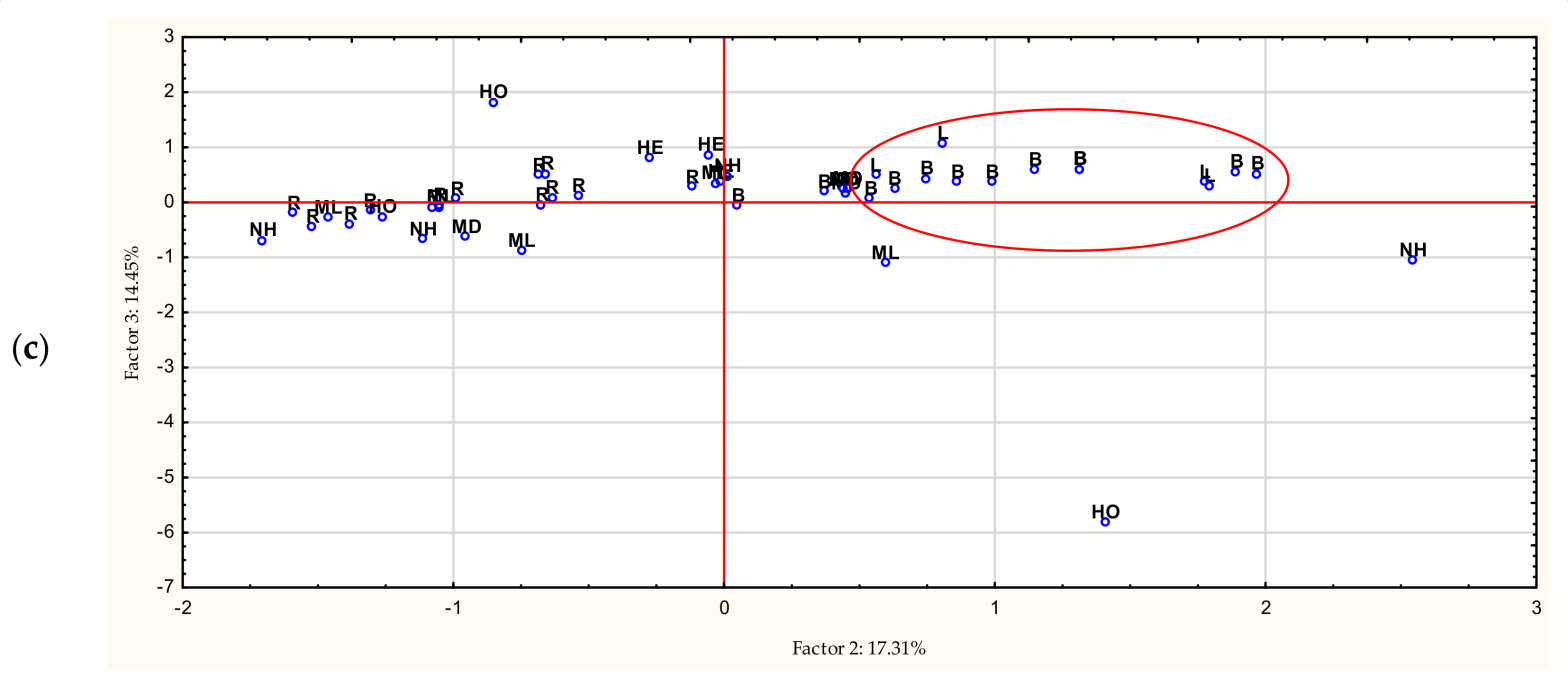
Projection of cases depending on the phenolic acids in a two – factor plane: factor 1 × factor 2 (**a**), factor 1 × factor 3 (**b**), factor 2 × factor 3 (**c**).

On the basis of the eigenvectors, it can be assessed that factor 1 is related to the following components: *p*-CA (0.44), 4-HBA (0.41), SA (−0.46), and CA (−0.47). The second component is related to 4-HBA (0.54) and *p*-CA (0.50), and the third component 3,4-DHBA (−0.79). Figure 5 shows 2W plots of factor coordinates of the variables. Points are significant factor loadings for individual components. The farther a given load is from the center of the circle, the greater the correlation of the variable with the factor axis. Figure 6 present 2W plots of cases depending on the phenolic acids.

## 4. Discussion

Natural bee honeys are characterized by a very rich composition, which determines their health-promoting properties. We have made an attempt to search for compounds that are characteristic of honey obtained in Poland. For rape, multifloral, nectar–honeydew, and honeydew honey, no characteristic phenolic compounds were found that could be considered determinants of the authenticity of these varieties. 

Taking into account other quality criteria, honeydew honeys are distinguished by having the highest median of electrical conductivity (1.181 mS × cm^−1^). 

Multifloral honeys are characterized by a complex composition, without the dominant presence of one plant, which may result in the lack of advantage of a specific phenolic acid. 

In addition, for rape honey, it may be necessary to establish a method with other acids. Moreover, rape honeys show the lowest electrical conductivity—the median was 0.242 mS × cm^−1^.

Buckwheat honeys from Poland show the darkest color, which results in the highest color value on the Pfund scale (median: 159.8 mm Pfund), the highest color intensity (2109.2 mAU), and the highest total phenolic content (196.59 mg GAE/100 g). Moreover, in these honeys, we showed the highest medians of 4-HBA (3.129 mg/100 g) and *p*-CA (0.804 mg/100 g). The studies conducted by Starowicz et al. (2021) [21] showed a lower value of TPC in honey of this variety (average: 141.1 mg GAE/100 g), while our research allowed us to conclude that the average value of this parameter is 182.60 ± 61.08 mg GAE/100 g.

Heather honeys were characterized by the highest median of one of the determined phenolic acids: SA (0.852 mg/100 g). Research by Ecem Bayram et al. (2020) [22] indicated that 3,4-DHBA is a characteristic compound for this variety of honey. SA was a relatively abundant compound (193.77 and 242.33 mg/L). TPC in this variety, in line with our results, was 91.78 ± 4.25 mg GAE/100 g, while the results published by Starowicz et al. (2021) [21] indicated a much higher content, at the level of 159.2 mg GAE/100 g. 

Linden honey, despite the low content of phenolic compounds (27.50 mg GAE/100 g), was surprisingly characterized by high contents of 3,4-DHBA (1.993 mg/100 g), CA (1.746 mg/100 g), SA (1.107 mg/100 g), VA (0.304 mg/100 g), and *t*-FA (1.954 mg/100 g). Dimitrova et al. (2007) [23] determined the content of, inter alia, phenolic acids in 49 honey samples. In the case of linden honey (*n* = 4), the authors provided only the maximum value of CA—this was 1.57 mg/kg. Our analysis showed about a 10 times higher content of this ingredient, at the level of 1.679 ± 0.338 mg/100 g, and the maximum value was 1.998 mg/100 g. The research carried out on linden honey from Turkey showed a characteristically high CA content (642.94 mg/L) [22], which was consistent with our observations (1.679 ± 0.338 mg/100 g). The content of SA was indicated only as of the maximum value (0.29 mg/kg)—in our study, the average content was 1.085 ± 0.276 mg/kg. The average VA content in honey of this variety was indicated by the authors at the level of 1.19 mg/kg, while our research indicated a value almost two times higher (0.312 mg/100 g). These results are slightly divergent due to the fact that the apiaries from which the honey was obtained differed in geographical location—in the case of Dimitrova et al. (2007) [23], these were honeys from producers from Denmark, France, Germany, Italy, the Netherlands, Portugal, United Kingdom, and Spain, while in our study, all honeys were from Poland. Our analyses also show the existence of many dependencies between the measured phenolic acids, as well as other quality parameters. A high positive correlation between the contents of VA and SA and between SA and CA may indicate the common presence of individual phenolic acids in nectar, which is particularly visible in the case of linden honey.

The content of phenolic acids such as 3,4-DHBA, 4-HBA, VA, SA, *p*-CA, FA, CA in buckwheat and heather honey from Poland can be compared with the results obtained by Jasicka-Misiak et al. in 2012 [24], including heather (*n* = 15) and buckwheat honey (*n* = 7). In this study, similar contents of 3,4-HBA were obtained: our research showed the content of this compound at the level of 1.403 ± 0.419 mg/100 g, while Jasicka-Misiak et al. [24] showed a level of about 1.228 mg/100 g (average content based on the determination of seven samples). The average VA content found in our study was approximately 10 times lower than in the study published by Jasicka-Misiak et al. [24]. The content of CA and SA in heather and buckwheat honey is low, in some cases below the detection limit, which is confirmed by both our research and the above-mentioned team of authors. According to our determinations, the p-CA content in buckwheat honey ranged from 0.558 to 1.004 mg/100 g, while the results obtained by Jasicka-Misiak et al. [24] were more divergent and indicated contents of 0.026 to 4.551 mg/100 g, and their average was almost three times higher. 

Buckwheat honey show the highest value of the TPC parameter. Numerous scientific publications indicate their rich composition; they are characterized, among others, by the presence of many volatile compounds, such as the occurrence of i.a. isovaleric acid in honey of this variety [25].

Searching for biomarkers of honey varieties is a task that has been carried out for over a dozen years [26]. For example, Cabras et al. (1999) [27] showed that the marker for strawberry honey is 2,5-dihydroxyphenylacetic acid, called homogentisic acid. Its content is around 378 ± 92 mg/kg. On the other hand, studies characterizing heather honey from Poland showed the presence of a less common compound: 4-hydroxy-3-(1-methylethyl) benzaldehyde [28]. Lumichrome is indicated as a honey marker for polish yellow sweet clover [29].

Literature data show that the phenolic acids contained in honey can penetrate lymphocytes and protect DNA from oxidative damage by scavenging hydrogen peroxide and chelating ferrous ions, as shown in studies on mice [30].

Single phenolic acids show very promising activities. For example, ferulic acid has been shown to have anti-inflammatory properties [31] and potential anti-cancer properties [32], protocatechuic acid has anti-viral properties [33], and *p*-coumaric antidiabetic and antihyperlipidemic properties [34]. The above examples show that bee honey, being a mixture of many compounds with antioxidant properties, may show multidirectional activity.

The research conducted by Wilczyńska et al. (2010) showed that buckwheat honeys can be characterized by up to 100.00% of free radical scavenging capacity. Heather honeys turned out to be even more effective—all tested samples were characterized by a result of 100%. The lowest capacity was recorded for acacia honeys—from 25.58 to 35.90%. In our study, the median for buckwheat honeys was 41.1%. Moreover, Wilczyńska et al. showed that, in buckwheat honeys, the highest value of TPC was recorded (180.07 mg GAE/100 g). Our research showed a median of 196.59, with the highest value being 241.87 mg GAE/100 g [35].

Another study published by Pentoś et al. (2020) aimed to compare selected antioxidant properties of honey from Poland with Manuka honey. It was shown that Manuka honey has a TPC value of 492.65 ± 1.32 mg GAE/100 g, while the honey from Poland with the highest value of this parameter was buckwheat honey (334.04 ± 1.26 mg GAE/100 g). The honey with the second-highest TPC value was heather honey (183.85 ± 1.27 mg GAE/100 g) [36].

Dżugan et al. (2017) assessed, inter alia, results obtained in the FRAP test by Polish honeys. The highest result was obtained by buckwheat honeys (3635.49 ± 1328.22 µmol TE/kg), followed by honeydew (2153.37 ± 663.92 µmol TE/kg), while the lowest result was found for rapeseed honeys (656.73 ± 119.40 TE/kg). Our results were presented in a different way, but the trend was similar—we obtained the following results: 0.402 ± 0.010, 0.323 ± 0.017, and 0.030 ± 0.012 µmol Fe^2+^/mL, respectively [7]. The studies by Beretta et al. (2005) also confirm that buckwheat and honeydew honeys are characterized by one of the highest results (800.7–23.8 and 772.0–21.5 µM) [16].

Our study has some limitations. We tested different amounts of honey samples belonging to a particular variety. This was due to the improper labeling of honey by beekeepers. Future research should be based on an even selection of the number of samples. It seems necessary to develop a method that will allow the determination of all phenolic acids present in honey—this will allow for the creation of detailed characteristics and the development of characteristic ranges of variability. Additionally, it seems necessary to characterize the varieties in terms of the content of individual flavonoids.

## 5. Conclusions

Phenolic acids can be considered markers of the authenticity of Polish honeybee varieties, in particular, syringic acid, vanillic acid, and coffee acid for linden honeys, *p*-coumaric acid and 4-hydroxybenzoic acid for buckwheat honeys, and vanillic acid for honeydew honeys. Moreover, buckwheat honeys show the highest median of the TPC parameter, which indicates a high content of phenolic compounds in the honeys of this variety. This variety of honey can be recommended to enrich the diet with antioxidant ingredients.

## Figures and Tables

**Table 1:** Characteristics of the developed method.

Compounds	RT	LOD (mg/100 g)	LOQ (mg/100 g)
3,4–DHBA	9.183	0.099	0.300
4–HBA	16.570	0.092	0.278
VA	20.284	0.089	0.271
CA	21.756	0.106	0.322
SA	23.886	0.147	0.445
*p*–CA	40.572	0.138	0.418
*t*–FA	50.040	0.084	0.255

3,4–DHBA—3,4-dihydroxybenzoic acid, 4–HBA—4-hydroxybenzoic acid, CA—caffeic acid, LOD—limit of detection, LOQ—limit of quantitation, *p*–CA—*p*-coumaric acid, RT—retention time, SA—syringic acid, *t*–PA—*trans*-ferulic acid, VA—vanillic acid.

**Table 2:** The percentage of honey with the correct and incorrect definitions of the variety.

Variety–Declarations of Beekeepers	The Number of Attempts Correctly Classified	The Number of Attempts Is Classified Incorrectly
buckwheat (*n* = 15)	12	3
dandelion (*n* = 4)	0	4
heather (*n* = 3)	3	0
honeydew (*n* = 3)	3	0
linden (*n* = 9)	4	5
multi-flower light (*n* = 3)	3	0
nectar–honeydew (*n* = 4)	3	1
rape (*n* = 8)	8	0

**Table 3:** The value of the parameters for individual varieties of honey.

Variety (Sign)		Colour Scale (mm Pfund)	Colour Intensity (mAU)	TPC (mg GAE/100 g)	Water Content (%)	Electrical Conductivity (mS × cm^−1^)	DPPH (% Free Radical Scavenging)	FRAP (Equivalent of µmol Fe^2+^/mL)
**Buckwheat** **(B)**	Av. ± SD Min-Max Med Q1-Q3	166.4 ± 29.4	1816.0 ± 688.0	182.60 ± 61.08	18.9 ± 0.5	0.400 ± 0.043	42.0 ± 4.5	0.402 ± 0.010
		125.8–218.5	711.0–2634.7	44.95–241.87	18.1–19.9	0.326–0.507	34.9–52.7	0.379–0.417
		159.8	2109.2	196.59	18.9	0.391	41.1	0.403
		147.9–189.0	1229.0–2291.8	142.29–236.63	18.4–19.3	0.380–0.416	39.8–44.0	0.398–0.409
**Heather** **(He)**		125.2 ± 14.8	575.8 ± 179.5	91.78 ± 4.25	19.2 ± 0.7	0.552 ± 0.027	46.4 ± 3.7	0.141 ± 0.002
		111.1–140.7	468.0–783.0	87.72–96.20	18.6–19.9	0.534–0.583	42.3–49.5	0.139–0.143
		124.0	476.3	91.42	19.0	0.538	47.5	0.140
		111.1–140.7	468.0–783.0	87.72–96-20	18.6–19.9	0.5334–0.583	42.3–49	0.139–0.143
**Honeydew** **(Ho)**		109.9 ± 95.9	587.1 ± 327.0	86.0 ± 55.3	16.3 ± 0.6	1.728 ± 1.072	58.6 ± 4.0	0.323 ± 0.017
		49.8–220.5	215.3–830.0	42.8–148.3	15.7–16.8	1.041–2.963	55.9–63.2	0.312–0.343
		59.5	716.0	67.07	16.4	1.181	56.7 * B	0.315
		49.8–220.5	215.3–830.0	42.78–148.30	15.7–16.8	1.041–1.922	55.9–63.2	0.312–0.343
**Linden** **(L)**		43.5 ± 19.6	84.0 ± 44.0	29.23 ± 10.60	16.7 ± 0.7	0.502 ± 0.104	58.6 ± 1.4	0.083 ± 0.012
		20.0–64.2	49.0–148.3	18.24–43.69	15.7–17.1	0.396–0.597	56.6–59.7	0.071–0.099
		44.9 ** B	69.3 *** B	27.50 ** B	16.9	0.508	59.0 ** B	0.081
		27.8–59.2	57.5–110.5	22.44–36.03	16.2–17.1	0.413–0.592	57.5–59.7	0.075–0.091
**Multifloral dark (Md)**		124.4 ± 25.6	1424.7 ± 803.1	187.6 ± 194.3	19.2 ± 0.8	0.416 ± 0.026	56.7 ± 2.8	0.218 ± 0.015
		91.9–154.1	280.0–2160.0	55.60–467.83	18.1–20.0	0.380–0.437	53.2–59.3	0.198–0.232
		125.8	1629.3	113.50	19.3	0.423	57.2 * B	0.221
		107.3–141.5	953.7–1895.7	56.37–318.85	18.7–19.7	0.397–0.435	54.4–59.0	0.206–0.230
**Multifloral light (Ml)**		33.3 ± 24.3	155.6 ± 90.9	32.04 ± 3.80	18.6 ± 0.7	0.431 ± 0.109	45.3 ± 6.5	0.052 ± 0.032
		1.0–64.7	64.0–272.0	28.86–38.26	18.0–19.3	0.308–0.584	37.4–52.8	0.014–0.090
		37.4 *** B	128.0 * B	30.85 ** B	18.1	0.452	46.8	0.062 ** B
		18.9–44.2	85.0–229.0	29.44–32.79	18.1–19.3	0.344–0.466	39.8–49.6	0.023–0.070
**Nectar–honeydew** **(Nh)**		115.2 ± 55.2	322.6 ± 231.7	57.08 ± 11.54	17.8 ± 1.7	0.641 ± 0.031	57.4 ± 4.2	0.205 ± 0.010
		75.4–192.6	93.7–623.0	47.20–70.74	16.5–20.3	0.609–0.670	52.4–61.8	0.197–0.219
		96.5	286.8	55.19	17.2	0.642	57.8 * B	0.203
		75.5–155.0	145.8–499.3	47.51–66.65	16.8–18.8	0.614–0.667	54.1–60.8	0.198–0.213
**Rape** **(R)**		81.47 ± 31.88	127.7 ± 48.69	33.18 ± 6.28	18.7 ± 0.8	0.284 ± 0.092	47.9 ± 5.7	0.030 ± 0.012
		17.5–125.6	62.0–231.0	20.40–43.94	17.7–20.6	0.169–0.449	37.8–58.7	0.012–0.058
		84.8 ** B	126.0 *** B	35.10 ** B	18.6	0.242 ** Ho, ** Nh	48.0	0.030 *** B
		66.4–98.8	86.0–150.3	30.17–36.74	18.1–19.1	0.215–0.352	45.6–52.3	0.022–0.035

* *p* < 0.05, ** *p* < 0.01, *** *p* < 0.001.

**Table 4:** The value of the phenolic acids for individual varieties of honey (mg/100 g).

Variety (Sign)		3,4-DHBA	4-HBA	CA	*p*-CA	VA	SA	*t*-FA
**Buckwheat** **(B)**	Av. ± SD Min-Max Med Q1-Q3	1.403 ± 0.419	3.203 ± 0.736	0.194 ± 0.073	0.784 ± 0.129	0.151 ± 0.043	0.186 ± 0.127	0.095 ± 0.050
		0.784–2.233	1.699–4.432	<LOD-0.325	0.558–1.004	<LOD-0.193	<LOD-0.329	<LOD-0.175
		1.421	3.129	0.207	0.804	0.165	<LOD	<LOD
		1.101–1.558	2.869–3.515	0.177–0.219	0.678–0.870	<LOD-0.180	<LOD-0.198	<LOD-0.152
**Heather** **(He)**		0.539 ± 0.056	0.895 ± 0.172	0.215 ± 0.025	0.386 ± 0.059	0.162 ± 0.143	0.860 ± 0.159	0.106 ± 0.092
		0.505–0.604	0.736–1.078	0.189–0.239	0.340–0.452	<LOD-0.273	0.705–1.023	<LOD-0.166
		0.509	0.873	0.216	0.367	0.211	0.852	0.152
		0.505–0.604	0.736–1.078	0.189–0.239	0.340–0.452	<LOD-0.273	0.705–1.023	<LOD-0.166
**Honeydew** **(Ho)**		7.646 ± 12.383	0.287 ± 0.090	0.252 ± 0.024	0.249 ± 0.089	0.368 ± 0.472	0.166 ± 0.183	0.475 ± 0.493
		0.354–21.944	0.184–0.348	0.225–0.268	0.171–0.346	0.133–0.913	<LOD-0.317	<LOD-0.985
		0.639	0.329	0.264	0.230	0.107	<LOD	0.442
		0.354–21.944	0.184–0.348	0.225–0.268	0.171–0.346	0.133–0.913	<LOD-0.317	<LOD-0.985
**Linden** **(L)**		2.064 ± 0.278	0.200 ± 0.051	1.679 ± 0.338	0.212 ± 0.211	0.312 ± 0.080	1.085 ± 0.276	1.973 ± 2.142
		1.818–2.454	0.152–0.271	1.227–1.998	<LOD-0.403	0.240–0.402	0.726–1.399	<LOD-3.982
		1.993	0.188 * B	1.746 ** B	0.221 * B	0.304 * B	1.107 * L	1.954
		1.872–2.257	0.165–0.235	1.427–1.931	0.151–0.392	0.245–0.380	0.910–1.259	0.123–3.822
**Multifloral dark** **(Md)**		0.680 ± 0.258	1.572 ± 0.964	0.261 ± 0.075	0.631 ± 0.325	0.108 ± 0.047	<LOD	0.633 ± 1.164
		0.443–1.045	0.185–2.235	0.190–0.334	0.144–0.804	<LOD-0.146	<LOD	<LOD-2.376
		0.615	1.934	0.260	0.789	0.128	<LOD	0.087
		0.517–0.842	0.917–2.227	0.197–0.325	0.463–0.800	<LOD-0.136	<LOD	<LOD-1.267
**Multifloral light** **(Ml)**		0.646 ± 0.551	0.191 ± 0.061	0.332 ± 0.333	0.357 ± 0.185	0.108 ± 0.070	0.178 ± 0.396	1.741 ± 2.479
		0.193–1.596	0.108–0.251	<LOD-0.885	0.158–0.235	<LOD-0.165	<LOD-0.885	0.094–5.654
		0.541	0.179 ** B	0.225	0.373	0.118	<LOD	0.155
		0.354–0.545	0.167–0.251	0.203–0.346	0.235–0.377	<LOD-0.145	<LOD	<LOD -2.782
**Nectar–honeydew** **(Nh)**		1.019 ± 0.849	0.235 ± 0.068	0.512 ± 0.667	0.299 ± 0.280	0.116 ± 0.033	0.410 ± 0.541	2.773 ± 4.607
		0.233–2.222	0.140–0.298	<LOD-1.493	0.154–0.717	<LOD-0.189	<LOD-1.141	0.090–9.656
		0.810	0.251 * B	0.278	0.178	0.107	0.249	0.673 * B
		0.488–1.550	0.193–0.277	0.134–0.891	0.174–0.457	<LOD-0.152	<LOD-0.819	0.193–5.353
**Rape** **(R)**		0.455 ± 0.379	0.256 ± 0.139	0.334 ± 0.245	0.262 ± 0.145	0.102 ± 0.068	0.267 ± 0.313	0.124 ± 0.060
		<LOD-1.482	0.109–0.484	0.177–0.870	0.155–0.581	<LOD-0.246	<LOD-0.847	<LOD-0.237
		0.350 ** L, ** B	0.174 *** B	0.227	0.242 *** B	0.114	0.165	0.128
		0.265–0.385	0.153–0.411	0.199–0.237	0.168–0.325	<LOD-0.127	<LOD-0.419	0.096–0.155

3,4-DHEA—3,4-dihydroxybenzoic acid, 4-HBA—4-hydroxybenzoic acid, <LOD—below the detection limit, CA—caffeic acid, *p*-CA—*p*-coumaric acid, SA—syringic acid, *t*-FA—*trans*-ferulic acid, VA—vanillic acid. * *p*< 0.05, ** *p* < 0.01, *** *p* < 0.001.

**Table 5:** Correlations between individual parameters (*p* <0.05).

Parameter 1	Parameter 2	r	*p*
Color in Pfund scale	Colour intensity	0.82	0.001
Color in Pfund scale	TPC	0.77	0.001
Color in Pfund scale	Diastase number	0.51	0.001
Color in Pfund scale	3,4-DHBA	0.75	0.001
Color in Pfund scale	SA	−0.33	0.021
Color in Pfund scale	*p*-CA	0.51	0.001
Color in Pfund scale	*t*-FA	−0.57	0.001
Color in Pfund scale	CA	−0.45	0.001
Colour intensity	TPC	0.90	0.001
Colour intensity	Diastase number	0.51	0.001
Colour intensity	Water	0.33	0.020
Colour intensity	4-HBA	0.84	0.001
Colour intensity	VA	−0.39	0.005
Colour intensity	SA	−0.45	0.001
Colour intensity	*p*-CA	0.68	0.001
Colour intensity	*t*-FA	−0.52	0.001
Colour intensity	CA	−0.46	0.001
DPPH	Water	−0.37	0.008
DPPH	*p*-CA	−0.35	0.01
DPPH	*t*-FA	0.45	0.001
TPC	Diastase number	0.58	0.001
TPC	3,4-DHBA	0.33	0.020
TPC	4-HBA	0.79	0.001
TPC	VA	−0.30	0.038
TPC	*p*-CA	0.60	0.001
TPC	*t*-FA	−0.57	0.001
TPC	CA	−0.31	0.001
Diastase number	4-HBA	0.56	0.001
Diastase number	*p*-CA	0.55	0.001
Water	Electrical conductivity	−0.37	0.009
Water	4-HBA	0.31	0.026
Water	VA	−0.37	0.009
Water	*p*-CA	0.32	0.023
Water	CA	−0.36	0.011
Electrical conductivity	3,4-DHBA	0.40	0.005
Electrical conductivity	VA	0.29	0.040
Electrical conductivity	CA	0.42	0.002
FRAP	Colour in Pfund scale	0.68	0.001
FRAP	Colour intensity	0.82	0.001
FRAP	TPC	0.82	0.001
FRAP	Diastase number	0.50	0.001
FRAP	Electrical conductivity	0.38	0.008
FRAP	3,4-DHBA	0.53	0.001
FRAP	4-HBA	0.73	0.001
FRAP	*p*-CA	0.58	0.001
FRAP	*t*-FA	−0.38	0.006
3,4-DHBA	SA	0.30	0.034
3,4-DHBA	CA	0.37	0.009
4-HBA	SA	−0.29	0.040
4-HBA	*p*-CA	0.82	0.001
4-HBA	*t*-FA	−0.46	0.001
4-HBA	CA	−0.36	0.011
VA	SA	0.60	0.000
VA	*p*-CA	−0.32	0.024
VA	CA	0.60	0.001
SA	*p*-CA	−0.31	0.028
SA	CA	0.51	0.001
p-CA	*t*-FA	−0.30	0.038
p-CA	CA	−0.29	0.040
t-FA	CA	0.47	0.001

3,4-DHEA—3,4-dihydroxybenzoic acid, 4-HBA—4-hydroxybenzoic acid, CA—caffeic acid, *p*-CA—*p*-coumaric acid, SA—syringic acid, *t*-FA—trans-ferulic acid, TPC—total phenolic content, VA—vanillic acid.

## Data Availability

Data is contained within the article.
